# Retraction: LncRNA XIST promotes the progression of laryngeal squamous cell carcinoma via sponging miR-125b-5p to modulate TRIB2

**DOI:** 10.1042/BSR-2019-3172_RET

**Published:** 2022-01-11

**Keywords:** LncRNA XIST, miR-125b-5p, TRIB2

This article is being retracted from *Bioscience Reports* at the request of the authors, as the results of the repeated experiments did not show the statistical differences seen in the original study. Hence, the authors of this study feel the results of this paper are unreliable.

The Editor-in-Chief and Editorial Board agree with the Retraction.

